# Burden of disease from inadequate water, sanitation and hygiene in low- and middle-income settings: a retrospective analysis of data from 145 countries

**DOI:** 10.1111/tmi.12329

**Published:** 2014-04-30

**Authors:** Annette Prüss-Ustün, Jamie Bartram, Thomas Clasen, John M Colford, Oliver Cumming, Valerie Curtis, Sophie Bonjour, Alan D Dangour, Jennifer De France, Lorna Fewtrell, Matthew C Freeman, Bruce Gordon, Paul R Hunter, Richard B Johnston, Colin Mathers, Daniel Mäusezahl, Kate Medlicott, Maria Neira, Meredith Stocks, Jennyfer Wolf, Sandy Cairncross

**Affiliations:** 1 Department of Public Health and Environment, World Health OrganizationGeneva, Switzerland; 2 Gillings School of Global Public Health, University of North Carolina at Chapel HillChapel Hill, NC, USA; 3 Rollins School of Public Health, Emory UniversityAtlanta, GA, USA; 4 School of Public Health, University of California, BerkeleyBerkeley, CA, USA; 5 London School of Hygiene and Tropical MedicineLondon, UK; 6 Centre for Research into Environment and Health, Aberystwyth UniversityAberystwyth, UK; 7 Norwich Medical School, University of East AngliaNorwich, UK; 8 Department of Environmental Health, Tshware University of TechnologyPretoria, South Africa; 9 EAWAG, Swiss Federal Institute of Aquatic Science and TechnologyDübendorf, Switzerland; 10 Department of Health Statistics and Information Systems, World Health OrganizationGeneva, Switzerland; 11 Swiss Tropical and Public Health InstituteBasel, Switzerland; 12 University of BaselBasel, Switzerland

**Keywords:** burden of disease, diarrhoea, water, sanitation, hygiene

## Abstract

**Objective:**

To estimate the burden of diarrhoeal diseases from exposure to inadequate water, sanitation and hand hygiene in low- and middle-income settings and provide an overview of the impact on other diseases.

**Methods:**

For estimating the impact of water, sanitation and hygiene on diarrhoea, we selected exposure levels with both sufficient global exposure data and a matching exposure-risk relationship. Global exposure data were estimated for the year 2012, and risk estimates were taken from the most recent systematic analyses. We estimated attributable deaths and disability-adjusted life years (DALYs) by country, age and sex for inadequate water, sanitation and hand hygiene separately, and as a cluster of risk factors. Uncertainty estimates were computed on the basis of uncertainty surrounding exposure estimates and relative risks.

**Results:**

In 2012, 502 000 diarrhoea deaths were estimated to be caused by inadequate drinking water and 280 000 deaths by inadequate sanitation. The most likely estimate of disease burden from inadequate hand hygiene amounts to 297 000 deaths. In total, 842 000 diarrhoea deaths are estimated to be caused by this cluster of risk factors, which amounts to 1.5% of the total disease burden and 58% of diarrhoeal diseases. In children under 5 years old, 361 000 deaths could be prevented, representing 5.5% of deaths in that age group.

**Conclusions:**

This estimate confirms the importance of improving water and sanitation in low- and middle-income settings for the prevention of diarrhoeal disease burden. It also underscores the need for better data on exposure and risk reductions that can be achieved with provision of reliable piped water, community sewage with treatment and hand hygiene.

## Introduction

Information on the burden of disease, its causes and prevention is fundamental to health policy. Among other things, an improved understanding of the disease burden and the relative contribution of key risks points towards opportunities for preventive action in a context of increasing healthcare costs (OECD 2013).

In recognition of the value of this information, several comprehensive disease burden studies, focusing mainly on diarrhoeal diseases, have been undertaken in recent decades (Murray & Lopez 1996; WHO 2002, 2004, 2009; Prüss-Ustün *et al*. 2008; Lim *et al*. 2012). These report important changes in the roles of various risk factors (Clasen *et al*. 2014).

Inadequate drinking water, sanitation and hygiene (WASH) are important risk factors, particularly in low-income settings. In 2011, an estimated 768 million people relied on ‘unimproved’ water supplies (as defined by the WHO/UNICEF Joint Monitoring Program for Water and Sanitation – JMP), which are thought to have high levels of pathogen contamination (WHO & UNICEF 2013a). Many more use sources that are classified as ‘improved’ but are still unsafe for consumption (Bain *et al*. 2014). More than 2.5 billion people lack access to an improved sanitation facility (WHO & UNICEF 2013a). Inadequate hand hygiene practices have been estimated to affect 80% of the population globally (Freeman *et al*. 2014b).

The health risks from inadequate WASH have been documented previously (Esrey *et al*. 1991; Fewtrell *et al*. 2005; Waddington *et al*. 2009). However, the unpublished review on which the 2010 Global Burden of Disease (GBD) study is based (Lim *et al*. 2012) departed from earlier reviews by finding no additional benefit from further improvements such as higher water quality or continuous piped supply over the exposure defined as using ‘other improved water supplies’ (Engell & Lim 2013). A more recent systematic review, however, is largely consistent with previous evidence (Wolf *et al*. 2014).

Estimating the impact of WASH on diarrhoeal diseases has commonly been assessed with comparative risk assessment methods (Ezzati *et al*. 2002; WHO 2004; Lim *et al*. 2012), although other methods such as population intervention models could also be considered (Clasen *et al*. 2014). Other diseases cannot currently be estimated with such methods due to insufficient evidence and require alternative approaches. As these would require considerable additional assessments and analyses, they are not analysed in detail in this article.

Accrual of substantive recent evidence, as well as trends in the total diarrhoea burden, justifies the revision of methods and estimates of the burden of diarrhoeal disease associated with inadequate WASH. While the estimate presented focuses mainly on low- and middle-income settings, the approach used can accommodate a wider range of settings. An overview of previous findings on the impacts of WASH on other diseases than diarrhoea is also provided.

## Methods

### Framework for estimation

For the purpose of this assessment, we defined WASH to include the following transmission pathways: (i) ingestion of water – for example diarrhoea, arsenicosis, fluorosis; (ii) lack of water linked to inadequate personal hygiene – for example diarrhoea, trachoma, scabies; (iii) poor personal, domestic or agricultural hygiene – for example diarrhoea, Japanese encephalitis; (iv) contact with water – for example schistosomiasis; (v) vectors proliferating in water – for example malaria; and (vi) contaminated water systems – for example legionellosis (Prüss *et al*. 2002). The impact of WASH on most diseases cannot be precisely estimated, because of insufficient information on global exposures of concern or lack of widely applicable risk estimates matching the exposures. Table 1 provides an overview of main diseases related to WASH and previously estimated attributable fractions by disease. An overview of previous results is provided in the Discussion section.

**Table 1:** Diseases related to water, sanitation and hygiene

Disease outcomes and range of the fraction of disease globally attributable to WASH^a^			
Contribution of WASH not quantified at global level	0–33%	33–66%	66–100%
Hepatitis A, E, F Legionellosis Scabies Arsenicosis Fluorosis Methaemoglobinaemia	Onchocerciasis	Lymphatic filariasis Malaria Undernutrition and its consequences Drowning	Ascariasis Hookworm Trichuriasis Dengue Schistosomiasis Japanese encephalitis Trachoma

WASH, water, sanitation and hygiene.

Includes diseases other than diarrhoea.

Adapted from: Prüss-Ustün and Corvalán (2007), Prüss-Ustün *et al*. (2008).

a Estimates based on previous assessments combining systematic literature reviews with expert opinion.

The burden of diarrhoea associated with inadequate WASH can, however, be estimated using comparative risk assessment methods (Ezzati *et al*. 2002; WHO 2004; Lim *et al*. 2012) and is addressed in detail in this article. This approach estimates the proportional reduction in disease or death that would occur if exposures were reduced to an alternative baseline level bearing a minimum risk (also referred to as theoretical minimum risk), while other conditions remain unchanged. It is derived from the proportion of people exposed to the conditions of interest and the relative risk of disease related to the exposure.

Proportion of the population exposed and relative risk values were specified by level of exposure, age group and sex. Estimates were calculated for the 145 low- and middle-income countries (WHO Member States with income levels defined by the World Bank for 2012), which were then grouped into the six WHO Regions (WHO 2013b, Supporting Information). The estimation was performed for the year 2012 (WHO 2013a).

### Selection of exposure-risk pairs for diarrhoeal disease

#### Water

Exposure levels were selected according to the availability of exposure data and corresponding exposure-risk information (Wolf *et al*. 2013, 2014) and included the following: (i) using an unimproved water source; (ii) using an improved water source other than piped to premises; (iii) using basic piped water on premises (improved source); and (iv) using a water filter or boiling water in the household (on water from an unimproved or improved source).

As piped water on premises is often intermittent and of suboptimal quality, the risks associated with having access to a ‘basic’ piped water supply in most settings of low- and middle-income countries are not equal to zero. A single study (meeting the criteria for the systematic review – Wolf *et al*. 2014) was identified which could inform this estimate of risk (i.e. by demonstrating the effect of improving water quality through the better operation of an existing piped water system in a context relevant to a low- or middle-income country). This study (Hunter *et al*. 2010) showed a significant and large reduction in diarrhoea and had an effect size of 0.32 (95% CI: 0.14–0.74). This evidence is also supported by information from disease outbreaks resulting from contaminated piped water (Mermin *et al*. 1999) and by interventions to further improve water supply systems in developed countries (Gunnarsdottir *et al*. 2012). However, given that only one study is currently available on the improvement beyond piped water to premises, a conservative approach was taken and the next best exposure level was used as the counterfactual (i.e. baseline) exposure (which consists of using a filter to treat water at household level – Wolf *et al*. 2014). Household water filtering is therefore used as a proxy for further improvement beyond currently available improved water sources.

It has been documented that lower water use (Cairncross & Feachem 1993; Royal Scientific Society 2013) and increasing distance to a water source (Tonglet *et al*. 1992; Galiani *et al*. 2007; Pickering & Davis 2012; Evans *et al*. 2013) have been associated with an increased risk of diarrhoea. The number of studies identified, however, was not sufficient to derive a pooled estimate. To account for this, in the current analysis, people living at distances greater than a 30-min round trip from their water source were assumed to have unimproved water.

Among assessed household water treatment methods, after adjusting for bias introduced through non-blinding of study participants, only use of a filter showed significant reductions in diarrhoeal disease morbidity; the effect of other methods, such as solar disinfection and chlorination, became non-significant after adjusting for bias (Wolf *et al*. 2014). Boiling of drinking water is a widespread practice in certain areas (Rosa & Clasen 2010), and while boiling may be an effective water treatment, recontamination has been reported (Clasen *et al*. 2008; Rosa *et al*. 2010). Only one study, however, has reported on the health effect of this practice (Iijima *et al*. 2001) and, for the purposes of this analysis, people who boil their drinking water have been classified with those who filter their water. Safe storage was assumed for all households filtering or boiling their water as information on recontamination was not available. Households filtering or boiling their water, with subsequent safe storage, represent the minimum risk group in this analysis.

**Figure 1:**
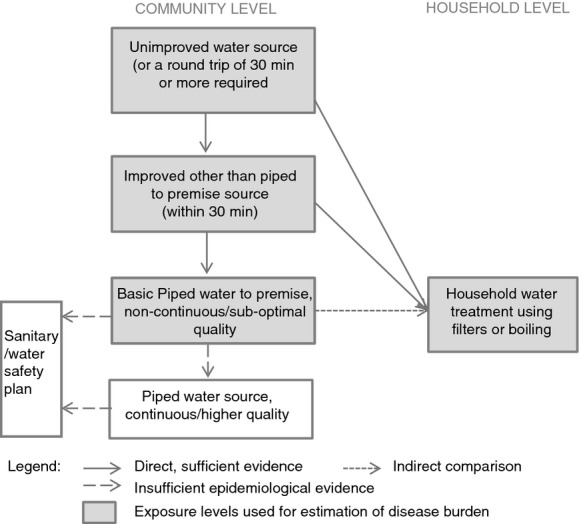
Exposure levels and associated risks for drinking water-related burden of disease estimates.

The exposure levels for inadequate drinking water, used in this analysis, along with additional levels of exposure to water with improved quality or quantity that are not currently supported by sufficient epidemiological evidence, are shown in Figure 1. This approach can accommodate further exposure levels when supported by sufficient evidence. The exposure–risk relationships (taken from Wolf *et al*. 2014) are summarised in Table 2.

**Table 2:** Effect sizes used for estimating diarrhoeal disease burden estimates from inadequate drinking water

Baseline water	Outcome water		
	Improved sourceother than piped to premise	Basic piped water to premise‡	Filter and safe storage in the household*
Unimproved source	0.89 (0.78–1.01)	0.77 (0.64–0.92)	0.55 (0.38–0.81)
Improved source other than piped to premise		0.86 (0.72–1.03)	0.62 (0.42–0.93)
Basic piped water to premise‡			0.72 (0.47–1.11)†

Not all steps of this body of evidence may be significant; however, risk estimates of the overall chain of improvements in water and sanitation are significant.

Adapted from: Wolf *et al*. (2014); Figures constitute relative risks (and 95% confidence intervals).

* Estimate for filtering water in the household also used for boiling water.

† Obtained through indirect comparison with improved non-piped or community water source in the meta-regression.

‡ possibly non-continuous, and/or of sub-optimal quality.

#### Sanitation

The only exposure levels for inadequate sanitation with both globally representative exposure data and sufficient evidence for its effect on diarrhoea were the use of an improved or unimproved sanitation facility (as defined by JMP – WHO & UNICEF 2013b). Evidence based on two studies suggests that further reduction in diarrhoea morbidity can be achieved with sewer connections in urban settings (although it should be noted that potential adverse impacts of untreated sewage on receiving communities have not been well studied). As the evidence for sewer connection was limited, it was not retained for the current diarrhoeal disease burden estimates. The overall effect for access to an improved sanitation facility on reduction in diarrhoea morbidity used was 28% (RR 0.72, 95% CI 0.59–0.88) from Wolf *et al*. (2014).

#### Hygiene

An updated review of the evidence linking interventions of the promotion of hand hygiene with soap and diarrhoea morbidity (Freeman *et al*. 2014b) showed a 40% reduction in diarrhoea (RR 0.60, 95% CI 0.53–0.68). When correcting for bias due to non-blinding in studies using subjective health outcomes (Savović *et al*. 2012), this estimate changes to 0.77 (95% CI 0.32–1.86) and becomes non-significant. It should be noted, however, that this bias correction is based on a wide array of medical interventions, which may be of limited applicability to this type of intervention. A 23% reduction in diarrhoeal disease risk remains the best estimate of the effect of handwashing promotion.

### Estimation of the proportion of people exposed

We drew on the definitions of the use of improved water sources, piped water to premises and improved sanitation of the JMP (WHO & UNICEF 2013b). Exposure by country was estimated by multilevel modelling as previously described (Wolf *et al*. 2013) based on over 1400 data points from national and international household surveys and censuses reported by JMP (WHO & UNICEF 2013b). Households with a travel time to the water source >30 min were deducted from improved sources at community level. We applied a linear two-level model with a logit transformation of the dependant variable (use of improved water source, improved sanitation or piped water to premises) to obtain estimates for the year 2012 (Wolf *et al*. 2013). The model also used a cubic spline transformation of the main predictor (time) and WHO region (WHO 2013b) as covariates, as well as a random intercept and slope by country.

Travel time of >30 min was reported by 178 household surveys [Demographic Health Surveys (USAID 2014), Multiple Indicator Cluster Surveys (UNICEF 2014), World Health Surveys (WHO 2014)] from 79 countries and was estimated for the year 2012 using a similar but simplified approach with a linear two-level model, with time and region as covariates and a random intercept and slope by country.

The proportion of country populations practising water treatment in the household was estimated using data from 78 household surveys [Demographic Health Surveys (USAID 2014), Multiple Indicator Cluster Surveys (UNICEF 2014), World Health Surveys (WHO 2014)] from 68 countries containing information on reported household water treatment (including chlorination, boiling, filtering, solar disinfection and others). A similar modelling approach as for travel time >30 min was used to obtain the proportion of households boiling or filtering their drinking water for the year 2012, with the difference that it did not use a random slope at country level. For countries with no information, the regional mean trend was taken as the best estimate.

Based on a review of water quality (Bain *et al*. 2014), no significant proportion of households in low- and middle-income settings are currently assumed to benefit from regulated and fully functional piped water supply systems.

The hand-washing prevalence, based on 75 observations, was taken from the systematic review reported by Freeman *et al*. (2014b).

### Population-attributable fractions of diarrhoeal disease for individual risk factor and for the cluster

For each risk factor, the population-attributable fraction (PAF) was estimated by comparing current exposure distributions to a counterfactual distribution, for each exposure level, sex and age group, and by country:
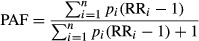
1where *p*_*i*_ and RR_*i*_ are the proportion of the exposed population and the relative risk at exposure level *i*, respectively, and *n* is the total number of exposure levels.

Exposure to inadequate WASH is related by similar mechanisms and policy interventions. The following formula has been proposed for the estimation of burden attributable to a cluster of risk factors (Lim *et al*. 2012):
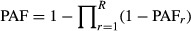
2where *r* is the individual risk factor and *R* the total number of risk factors accounted for in the cluster. This formula assumes that risk factors are independent. This assumption is likely to be an oversimplification for WASH as, for instance, handwashing promotion is unlikely to be effective if water quantity is limited. However, this approach has been applied in the assessment for ease of interpretation of the results, and in the absence of a more suitable approach.

### Estimation of burden of diarrhoeal disease

The burden of disease attributable to each risk factor (AB), or to the cluster of risk factors, in deaths or disability-adjusted life years (DALYs), was obtained by multiplying the PAFs by the total burden of disease of diarrhoea (B):
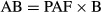
3

The PAFs were applied equally to burden of disease in deaths and DALYs, and we assumed that the case fatality related to WASH was the same as the mean case fatality of diarrhoeal diseases.

### Uncertainty estimates

To estimate uncertainty intervals, we developed a Monte Carlo simulation of the results with 5000 draws of the exposure distribution, and of the relative risks. As lower and upper uncertainty estimates, we used the 2.5 and 97.5 percentiles of the attributable fractions, attributable deaths and DALYs resulting from the Monte Carlo analysis (Palisade 2013).

## Results

The worldwide distribution of exposure and the resulting attributable deaths and DALYs from diarrhoeal disease associated with inadequate WASH practices were estimated for the year 2012.

### Exposure estimates

In low- and middle-income countries, it was found that in 31% of households people report boiling or filtering their water; 31% of households use piped water to premises; 27% use a non-piped or community water source; 12% use only an unimproved water source and do not filter or boil their water; and on the sanitation side, 58% of households were estimated to use an improved sanitation facility, respectively.

Handwashing after using a sanitation facility or contact with faecal material is practised by 19% of people worldwide (based on observation data), with a mean of 14% in low- and middle-income countries, and 43% in high-income countries (Freeman *et al*. 2014b). The estimated regional distribution of exposure is presented in Table 3 (drinking water) and Table 4 (sanitation and hygiene); more detail by country is provided in the Supporting Information.

**Table 3:** Distribution of the population to exposure levels of drinking water, by region, for 2012

Region	Use of piped water on premises		Use of non-piped or community sources		Use of unimproved water sources		Total*
	Proportion of total population by region						
Filtering/boiling in the household	Without	With	Without	With	Without	With	
Sub-Saharan Africa	0.16	0.03	0.36	0.04	0.38	0.04	1.00
America, LMI	0.58	0.30	0.05	0.01	0.05	0.01	1.00
Eastern Mediterranean, LMI	0.54	0.04	0.25	0.01	0.15	0.01	1.00
Europe, LMI	0.54	0.27	0.10	0.05	0.03	0.02	1.00
South-East Asia	0.16	0.09	0.48	0.14	0.09	0.04	1.00
Western Pacific, LMI	0.31	0.35	0.13	0.14	0.04	0.04	1.00
Total LMI	0.31	0.18	0.27	0.09	0.12	0.03	1.00

LMI, low and middle income.

* The total may not equal the sum of numbers displayed in the rows due to rounding error.

**Table 4:** Distribution of the population to exposure levels of sanitation and hygiene, by region, for 2012

Region	Access to improved sanitation facility	Prevalence of handwashing after contact with excreta
	Proportion of total population	
Sub-Saharan Africa	0.35	0.14
America, HI	–	0.49
America, LMI	0.83	0.16
Eastern Mediterranean, HI	–	0.44
Eastern Mediterranean, LMI	0.68	0.14
Europe, HI	–	0.44
Europe, LMI	0.87	0.15
South-East Asia	0.47	0.17
Western Pacific, HI	–	0.43
Western Pacific, LMI	0.64	0.13
Total	–	0.19
Total HI	–	0.43
Total LMI	0.58	0.14

LMI, low and middle income; HI, high income; –, not estimated.

### Estimates of the burden of diarrhoeal disease

The resulting burden of diarrhoea, in low- and middle-income countries, linked to these exposures amounts to 502 000 deaths associated with inadequate water and 280 000 deaths due to inadequate sanitation from a total of 1.50 million diarrhoeal deaths in the year 2012.

In addition, it was estimated that 297 000 deaths could be prevented by the promotion of hand hygiene, although this estimate is based on an effect size which is not statistically significant. The estimate without adjusting for non-blinding would be 539 000 deaths.

Together (using Equation 2), the deaths attributable to inadequate water and sanitation amount to 685 000. Adding (bias-adjusted) inadequate hand hygiene increases this estimate to 842 000 deaths, which represents 1.5% of the global disease burden in 2012. A regional summary of attributable deaths and DALYs for each of the risk factors is provided in Tables 7, and the cluster data are shown in Table 8. Detail by country can be found in the Supporting Information.

**Table 5:** Diarrhoea burden attributable to inadequate water by region, 2012

Region	PAF (95% CI)	Deaths (95% CI)	DALYs (in 1000s) (95% CI)
Sub-Saharan Africa	0.38 (0.19–0.50)	229 316 (106 664–300 790)	17 587 (8152–23 065)
America, LMI	0.26 (0.14–0.33)	6441 (624–9748)	522 (39–801)
Eastern Mediterranean, LMI	0.36 (0.19–0.46)	50 409 (22 498–66 604)	4046 (1784–5351)
Europe, LMI	0.16 (0.10–0.26)	1676 (196–2606)	174 (19–271)
South-East Asia	0.32 (0.11–0.44)	207 773 (59 708–293 068)	10 748 (3097–15 160)
Western Pacific, LMI	0.20 (0.09–0.27)	6448 (2005–9469)	716 (198–1081)
Total LMI	0.34 (0.16–0.45)	502 061 (217 119–671 945)	33 793 (14 930–44 871)

DALYs, disability-adjusted life years; PAF, population-attributable fraction; LMI, low and middle income.

**Table 6:** Diarrhoea burden attributable to inadequate sanitation by region, 2012

Region	PAF (95% CI)	Deaths (95% CI)	DALYs (in 1000s) (95% CI)
Sub-Saharan Africa	0.21 (0.07–0.31)	126 294 (42 881–186 850)	9694 (3291–14 333)
America, LMI	0.09 (0.03–0.15)	2370 (774–3724)	188 (61–295)
Eastern Mediterranean, LMI	0.17 (0.06–0.26)	24 441 (8339–36 809)	1914 (651–2887)
Europe, LMI	0.03 (0.01–0.06)	352 (107–597)	36 (11–61)
South-East Asia	0.19 (0.06–0.28)	123 279 (42 116–185 426)	6376 (2177–9595)
Western Pacific, LMI	0.11 (0.04–0.17)	3709 (1171–5954)	444 (136–737)
Total LMI	0.19 (0.07–0.29)	280 443 (95 699–417 482)	18 650 (6380–27 769)

DALYs, disability-adjusted life years; PAF, population-attributable fraction; LMI, low and middle income.

**Table 7:** Diarrhoea burden attributable to inadequate hand hygiene by region, 2012

Region	PAF (95% CI)	Deaths (95% CI)	DALYs (in 1000s) (95% CI)
Sub-Saharan Africa	0.20 (0–0.61)	122 955 (0–365 911)	9411 (0–28 006)
America, HI	0.13 (0–0.45)	–	–
America, LMI	0.20 (0–0.60)	5026 (0–15 013)	416 (0–1243)
Eastern Mediterranean, HI	0.14 (0–0.48)	–	–
Eastern Mediterranean, LMI	0.21 (0–0.61)	28 699 (0–85 369)	2314 (0–6884)
Europe, HI	0.14 (0–0.48)	–	–
Europe, LMI	0.19 (0–0.59)	1972 (0–5975)	202 (0–611)
South-East Asia	0.20 (0–0.60)	131 519 (0–392 018)	6857 (0–20 444)
Western Pacific, HI	0.16 (0–0.50)	–	–
Western Pacific, LMI	0.21 (0–0.61)	6690 (0–19 891)	758 (0–2253)
Total	0.20 (0–0.60)	–	–
Total HI	0.14 (0–0.47)	–	–
Total LMI	0.20 (0–0.60)	296 860 (0–885 355)	19 958 (0–59 491)

DALYs, disability-adjusted life years; PAF, population-attributable fraction; LMI, low and middle income; HI, high income; –, not estimated.

**Table 8:** Diarrhoea deaths attributable to the cluster of inadequate water, and inadequate sanitation and hand hygiene

Region	Inadequate water, sanitation and hand hygiene		Inadequate water and sanitation	
	PAF (95% CI)	Deaths (95% CI)	PAF (95% CI)	Deaths (95% CI)
Sub-Saharan Africa	0.61 (0.55–0.66)	367 605 (326 795–402 438)	0.51 (0.47–0.55)	307 493 (276 989–335 899)
America, LMI	0.46 (0.36–0.50)	11 519 (9310–13 616)	0.32 (0.28–0.34)	8125 (7101–9158)
Eastern Mediterranean, LMI	0.58 (0.47–0.66)	81 064 (65 359–94 707)	0.47 (0.40–0.53)	65 700 (55 266–75 876)
Europe, LMI	0.35 (0.28–0.46)	3564 (2462–4678)	0.19 (0.19–0.27)	1970 (1654–2280)
South-East Asia	0.56 (0.36–0.70)	363 904 (225 359–477 720)	0.45 (0.31–0.57)	291 763 (193 198–383 423)
Western Pacific, LMI	0.44 (0.31–0.54)	14 160 (10 035–18 009)	0.29 (0.23–0.33)	9429 (7519–11 242)
Total LMI	0.58 (0.48–0.65)	841 818 (699 059–963 626)	0.47 (0.40–0.53)	684 479 (580 456–780 463)

PAF, population-attributable fraction; LMI, low and middle income.

Among children under 5 years, 361 000 deaths could have been prevented through reduction of these risks in low- and middle-income settings, representing 5.5% of the total burden of disease in this age group.

## Discussion

These estimates of the burden of diarrhoea attributable to inadequate WASH are lower than previous estimates coordinated by WHO (WHO 2009) and higher than the recent estimate of the 2010 GBD study (Lim *et al*. 2012). There is strong evidence that the number of deaths due to diarrhoeal disease has dropped considerably since 2004 (WHO 2009; Liu *et al*. 2012; Lozano *et al*. 2012) due to a combination of improved management of diarrhoeal disease (especially the use of oral rehydration therapy) and better access to water and sanitation. This is in line with the lower burden of diarrhoeal disease estimates in both the 2010 GBD study and the current work. The larger burden of diarrhoeal disease found in this study, compared with the 2010 GBD study, can be explained by the different counterfactuals used, the consideration in this study of disease burden due to poor hand hygiene and to the adjustments made to account for bias resulting from the lack of blinding in studies on different household water treatment interventions.

The estimate of diarrhoeal disease burden attributable to inadequate WASH practices is limited by the underlying evidence, which remains scarce for the transition between an improved water source and a functional and regulated water supply system. The evidence is also limited on sanitation; in particular, there is a dearth of information on wastewater and excreta management from improved facilities and the impact this has on downstream communities when it is disposed of, untreated, to the environment. In addition, a conservative effect size was chosen for the impact of hand hygiene on diarrhoea, based on figures adjusted for possible bias (Freeman *et al*. 2014b). This approach is, thus, more conservative than previous estimates (Curtis & Cairncross 2003).

Exposure data are limited in terms of representative measures of water quality. Handwashing prevalence has not yet been widely assessed, although studies have shown surprisingly little variation across countries and population groups within income groups (Freeman *et al*. 2014b). Surveys reporting the use of household water treatment options have shown some over-reporting. This would, however, have led to an underestimation of diarrhoeal disease burden in this analysis as households reported as filtering or boiling their water were assigned as having no risk related to inadequate WASH.

Certain potentially relevant exposure/exposure-risk pairs cannot yet be considered. These include, for example, incomplete community sanitation (i.e. incomplete community coverage) meaning that contact with excreta may persist within the community. Another example consists in improved sanitation facilities without treatment, which are likely to result in exposure of receiving communities to untreated sewage and could affect 22% of the global population (Baum *et al*. 2013). Also, this assessment is limited to non-outbreak situations.

The global assessment of exposure to faecal contamination through drinking water (Bain *et al*. 2014) has highlighted that piped water supplies in the American, European and Western Pacific low- and middle-income regions show particularly low contamination in urban areas, with <10% of investigated samples faecally contaminated. The relative risks from the meta-regression (Wolf *et al*. 2014) may overrate the risks of water sources with such low proportions of contamination, as they have been relatively poorly investigated in the underlying epidemiological literature. If assuming that urban piped supplies in those regions carry no increased risk for diarrhoea, the total diarrhoea burden from inadequate water sources would have decreased from 502 000 to 497 000 deaths in 2012, with 2800 fewer deaths in the American region, 700 fewer deaths in the European region and 1500 fewer deaths in the Western Pacific region, respectively. The contamination of piped water in those regions may, however, have been underestimated because (i) studies tend to take place in formal urban areas and especially in capital cities, (ii) the assessment reported the per cent of samples containing contamination rather than compliance with WHO guidelines, and (iii) the focus was on water quality at the source and not stored at home or sampled just before consumption (Bain *et al*. 2014).

The current estimation has focused on diarrhoeal diseases and has not re-analysed the impact on other diseases, which have been linked to inadequate WASH, including soil-transmitted helminth infections (Ziegelbauer *et al*. 2012), vector-borne diseases (Emerson *et al*. 2000), environmental enteropathy (Humphrey 2009). Furthermore, improved WASH has been shown to significantly reduce undernutrition (Dangour *et al*. 2013), a major cause of mortality in children under 5 years of age (Black *et al*. 2013). Previous estimates, based on literature reviews combined with expert opinion, have, however, attempted to provide quantitative estimates of other diseases than diarrhoea, with the following results: In 2004, 881 000 deaths were attributed to water supply, sanitation and hygiene, mainly through the effect on undernutrition and its consequences, but also from schistosomiasis and lymphatic filariasis. The impacts of water resource management, mainly on malaria but also dengue and Japanese encephalitis, were estimated to amount to 557 000 deaths in the same year. Finally, safer water environments could have prevented 244 000 deaths from drowning, globally (Prüss-Ustün & Corvalán 2007; Prüss-Ustün *et al*. 2008). Although these figures would require an update, they indicate that the impacts of WASH on other diseases combined are likely to be even higher than those on diarrhoea.

The estimation of diarrhoeal disease burden relies on proxies such as access to water and sanitation facilities rather than water quality, water quantity or behaviours associated with these facilities (such as consistent or exclusive use by individuals) which are also a determining factor in characterising actual exposure. They were selected because of the available exposure information and their best match in the latest findings on risk estimates from the epidemiological literature. Greater precision of estimates is expected with better assessment of these more proximal risks and their population exposures. In addition, in common with a number of other disease burden estimates (Lim *et al*. 2012), the estimate is based on risk estimates for morbidity rather than mortality.

Due to these limitations, it is unlikely that this estimate accounts for the full health benefits in diarrhoea reduction that could be achieved by improvements in WASH. By relying on evidence of interventions that have often only achieved limited or partial compliance, this disease burden reflects reduction in diarrhoea that can be achieved with currently documented interventions in low- and middle-income countries. It is unlikely that the estimate accounts for the full reduction in burden that could be achieved by well-functioning water supply or sewage systems. For example, this estimate does not reflect health benefits that may be achieved through improvements following the implementation of management systems such as water safety plans (Gunnarsdottir *et al*. 2012), a proactive, comprehensive approach to managing risks throughout the water supply system. In addition, the estimates do not account for the potential impact of improvements to institutional settings, such as health centres and schools, and where studies have shown impact on other age groups (Dreibelbis *et al*. 2014; Freeman *et al*. 2014a).

Through the reassessment of the evidence linking drinking water to diarrhoea using a more scaled approach (Wolf *et al*. 2014), it has been possible to develop an estimate that takes account of the reduction in risks when further improving water quality or quantity over what is currently defined as an ‘improved source’, which was not carried out in more basic assessments (Lim *et al*. 2012). Indeed, improved water sources have been shown to carry important contamination and risks to a significant share of the population (Bain *et al*. 2014).

The separate assessment of the risks of WASH is not ideal, as those risk factors are likely to have linkages in terms of both exposure and effects on diarrhoeal risk. This choice was made, however, to facilitate policy interpretation, and because of the availability of factor-specific data sets. Nevertheless, the validity of some of these aspects, such as joint interventions, has been assessed in the meta-regression (Wolf *et al*. 2014) by testing the significance of covariates.

It is acknowledged that this assessment does not account for a number of relevant exposures including access to a continuous supply of safe piped water, community sewerage which prevents exposure to untreated wastewater or excreta (rather than focusing on household exposure alone) – evidence in this area is still limited. The counterfactual for the current assessment corresponds to currently achievable options that have been documented in developing countries and does not yet take into account the improvements that could be made beyond such a status. Although this assessment is limited to low- and middle-income settings, it is acknowledged that health risks exist even in apparently well-managed drinking water systems in developed countries (Zmirou *et al*. 1995; Naumova *et al*. 2005; Lake *et al*. 2007; Tinker *et al*. 2009), and further improvements have been shown to reduce health risks (Gunnarsdottir *et al*. 2012). This assessment does, however, act as a step towards a more comprehensive future estimate.

## Conclusion

This updated estimate of the diarrhoeal disease burden due to inadequate WASH has made use of a meta-regression approach to the evidence, based on specific information of baseline and outcome situation for each relevant study. This approach has resulted in a more refined estimate of disease burden according to exposure specificities. It can accommodate further consolidation as evidence accrues. It confirms the important role of the provision of safe water, adequate sanitation and hygiene promotion to protect health. Previous finding indicating an important impact of WASH on other diseases than diarrhoea further strengthens these findings.

## Supporting Information

Table S1 Deaths attributable to inadequate water, sanitation, and hygiene by low- and middle-income countriesa for the year 2012. **Table S2.** Deaths attributable to the combined inadequate water and sanitation, and to the combined inadequate water, sanitation and hygiene by low- and middle-income countrya, for the year 2012.
