# Supplementary material for: Burden of disease from inadequate water, sanitation and hygiene in low- and middle-income settings: a retrospective analysis of data from 145 countries
Source: Trop Med Int Health. 2014 Apr 30;19(8):894–905. doi: 10.1111/tmi.12329 (PMC4255749; doi:10.1111/tmi.12329)
Supplement: Table S1 — Deaths attributable to inadequate water, sanitation, and hygiene by low- and middle-income countriesa for the year 2012. Table S2. Deaths attributable to the combined inadequate water and sanitation, and to the combined inadequate water, sanitation and hygiene by low- and middle-income countrya, for the year 2012. [file tmi0019-0894-SD1.docx]

*Journal of Tropical Medicine and International Hygiene*

**Supplemental Material**

**Burden of disease from water, sanitation and hygiene in low resource settings: a retrospective analysis of data from 145 countries**

Annette Prüss-Ustün, Jamie Bartram, Thomas Clasen, John M. Colford, Jr., Oliver Cumming, Valerie Curtis, Sophie Bonjour, Alan D. Dangour, Jennifer De France, Lorna Fewtrell, Matthew C. Freeman, Bruce Gordon, Paul R. Hunter, Richard B. Johnston, Colin Mathers, Daniel Mäusezahl, Kate Medlicott, Maria Neira, Meredith Stocks, Jennyfer Wolf, Sandy Cairncross

Supplemental Material, Table 1. Deaths attributable to inadequate water, sanitation, and hygiene in low- and middle-income countries ^a^ for the year 2012, by country.

|  |  | **Unsafe water deaths** | | **Unsafe sanitation deaths** | | **Unsafe hygiene deaths** | |
| --- | --- | --- | --- | --- | --- | --- | --- |
| **Region^b^** | **Country** | **Mean** | **(95% CI)** | **Mean** | **(95% CI)** | **Mean** | **(95% CI)** |
| EmrLMI | Afghanistan | 6 420 | (3086, 8425) | 3 127 | (1062, 4791) | 4 639 | (0, 13947) |
| EurLMI | Albania | 5 | (0, 7) |  | (0, 0) | 2 | (0, 6) |
| AfrLMI | Algeria | 577 | (13, 892) | 62 | (18, 114) | 348 | (0, 1042) |
| AfrLMI | Angola | 13 634 | (6812, 18163) | 7 529 | (2565, 11302) | 7 404 | (0, 22142) |
| AmrLMI | Antigua and Barbuda | 0 | (0, 0) | 0 | (0, 0) | 1 | (0, 3) |
| AmrLMI | Argentina | 149 | (0, 270) | 10 | (0, 23) | 201 | (0, 621) |
| EurLMI | Armenia | 20 | (0, 34) | 2 | (1, 4) | 10 | (0, 29) |
| EurLMI | Azerbaijan | 88 | (29, 134) | 32 | (9, 57) | 141 | (0, 419) |
| SearLMI | Bangladesh | 5 784 | (1404, 8139) | 2 288 | (747, 3576) | 8 264 | (0, 24756) |
| EurLMI | Belarus | 10 | (0, 18) | 1 | (0, 2) | 46 | (0, 138) |
| AmrLMI | Belize | 2 | (0, 3) |  | (0, 1) | 4 | (0, 13) |
| AfrLMI | Benin | 1 877 | (866, 2486) | 1 191 | (423, 1730) | 1 499 | (0, 4483) |
| SearLMI | Bhutan | 23 | (6, 36) | 17 | (6, 27) | 51 | (0, 156) |
| AmrLMI | Bolivia | 354 | (8, 565) | 229 | (75, 356) | 286 | (0, 884) |
| EurLMI | Bosnia and Herzegovina | 2 | (0, 3) | 0 | (0, 0) | 2 | (0, 6) |
| AfrLMI | Botswana | 106 | (11, 160) | 42 | (13, 68) | 83 | (0, 247) |
| AmrLMI | Brazil | 1 137 | (0, 1994) | 326 | (99, 542) | 921 | (0, 2872) |
| EurLMI | Bulgaria | 3 | (0, 6) | 0 | (0, 0) | 6 | (0, 17) |
| AfrLMI | Burkina Faso | 3 814 | (1712, 5089) | 2 453 | (868, 3579) | 3 391 | (0, 10028) |
| AfrLMI | Burundi | 4 188 | (1925, 5528) | 1 726 | (557, 2720) | 2 157 | (0, 6451) |
| WprLMI | Cambodia | 377 | (159, 536) | 317 | (109, 476) | 884 | (0, 2622) |
| AfrLMI | Cameroon | 5 589 | (2624, 7373) | 2 374 | (777, 3679) | 4 115 | (0, 12307) |
| AfrLMI | Cape Verde | 12 | (4, 17) | 8 | (3, 11) | 26 | (0, 79) |
| AfrLMI | Central African Republic | 2 957 | (1308, 3913) | 1 510 | (508, 2311) | 1 383 | (0, 4136) |
| AfrLMI | Chad | 6 758 | (3211, 8892) | 4 312 | (1532, 6235) | 2 793 | (0, 8352) |
| AmrLMI | Chile | 54 | (0, 93) | 2 | (1, 4) | 25 | (0, 89) |
| WprLMI | China | 2 015 | (276, 3256) | 1 402 | (374, 2509) | 1 771 | (0, 5254) |
| AmrLMI | Colombia | 158 | (0, 267) | 47 | (14, 80) | 176 | (0, 545) |
| AfrLMI | Comoros | 117 | (42, 163) | 65 | (22, 99) | 86 | (0, 258) |
| AfrLMI | Congo | 1 206 | (590, 1607) | 764 | (269, 1129) | 793 | (0, 2371) |
| WprLMI | Cook Islands | 0 | (0, 0) | 0 | (0, 0) | 0 | (0, 1) |
| AmrLMI | Costa Rica | 18 | (0, 31) | 2 | (1, 3) | 12 | (0, 38) |
| AfrLMI | Côte d'Ivoire | 4 921 | (2181, 6601) | 3 121 | (1089, 4613) | 2 886 | (0, 8630) |
| AmrLMI | Cuba | 49 | (4, 77) | 7 | (2, 12) | 36 | (0, 110) |
| SearLMI | DPR Korea | 146 | (0, 263) | 67 | (19, 119) | 239 | (0, 725) |
| AfrLMI | DR Congo | 43 213 | (20522, 57039) | 22 779 | (7701, 34948) | 26 610 | (0, 79577) |
| EmrLMI | Djibouti | 131 | (33, 189) | 55 | (16, 90) | 121 | (0, 365) |
| AmrLMI | Dominica | 0 | (0, 0) | 0 | (0, 0) | 1 | (0, 3) |
| AmrLMI | Dominican Republic | 112 | (27, 168) | 26 | (8, 44) | 161 | (0, 499) |
| AmrLMI | Ecuador | 147 | (0, 241) | 23 | (6, 44) | 120 | (0, 371) |
| EmrLMI | Egypt | 779 | (0, 1311) | 39 | (10, 74) | 785 | (0, 2359) |
| AmrLMI | El Salvador | 80 | (10, 124) | 30 | (9, 50) | 86 | (0, 265) |
| AfrHI | Equatorial Guinea | 272 | (128, 361) | 102 | (33, 162) | 195 | (0, 583) |
| AfrLMI | Eritrea | 1 237 | (600, 1629) | 800 | (286, 1166) | 630 | (0, 1885) |
| AfrLMI | Ethiopia | 17 019 | (7877, 22307) | 9 367 | (3248, 13904) | 11 186 | (0, 33763) |
| WprLMI | Fiji | 12 | (1, 20) | 3 | (0, 8) | 17 | (0, 50) |
| AfrLMI | Gabon | 259 | (98, 361) | 148 | (49, 227) | 196 | (0, 587) |
| AfrLMI | Gambia | 242 | (103, 328) | 82 | (26, 133) | 201 | (0, 602) |
| EurLMI | Georgia | 5 | (0, 8) | 0 | (0, 1) | 16 | (0, 47) |
| AfrLMI | Ghana | 2 853 | (1082, 3870) | 1 886 | (665, 2739) | 2 446 | (0, 7234) |
| AmrLMI | Grenada | 1 | (0, 1) |  | (0, 0) | 1 | (0, 4) |
| AmrLMI | Guatemala | 718 | (0, 1173) | 204 | (62, 346) | 325 | (0, 1005) |
| AfrLMI | Guinea | 2 713 | (1178, 3647) | 1 726 | (617, 2530) | 1 848 | (0, 5525) |
| AfrLMI | Guinea-Bissau | 488 | (213, 647) | 289 | (102, 424) | 426 | (0, 1274) |
| AmrLMI | Guyana | 19 | (3, 28) | 4 | (1, 6) | 20 | (0, 63) |
| AmrLMI | Haiti | 1 770 | (806, 2354) | 933 | (314, 1462) | 553 | (0, 1712) |
| AmrLMI | Honduras | 333 | (0, 544) | 99 | (31, 164) | 99 | (0, 305) |
| SearLMI | India | 193 517 | (60971, 273518) | 115 404 | (39429, 173317) | 138 026 | (0, 420294) |
| SearLMI | Indonesia | 1 648 | (622, 2645) | 3 295 | (1045, 5225) | 7 543 | (0, 22897) |
| EmrLMI | Iran | 432 | (0, 729) | 37 | (9, 75) | 561 | (0, 1688) |
| EmrLMI | Iraq | 817 | (77, 1229) | 113 | (28, 216) | 637 | (0, 1916) |
| AmrLMI | Jamaica | 28 | (7, 41) | 7 | (2, 12) | 35 | (0, 109) |
| EmrLMI | Jordan | 39 | (0, 67) | 1 | (0, 2) | 46 | (0, 139) |
| EurLMI | Kazakhstan | 78 | (16, 126) | 6 | (2, 11) | 153 | (0, 455) |
| AfrLMI | Kenya | 7 735 | (3697, 10475) | 4 864 | (1658, 7280) | 4 531 | (0, 13873) |
| WprLMI | Kiribati | 7 | (3, 10) | 6 | (2, 9) | 3 | (0, 10) |
| EurLMI | Kyrgyzstan | 59 | (15, 88) | 5 | (1, 10) | 50 | (0, 148) |
| WprLMI | Lao PDR | 379 | (157, 568) | 273 | (87, 448) | 298 | (0, 884) |
| EurLMI | Latvia | 1 | (0, 1) | 0 | (0, 0) | 0 | (0, 1) |
| EmrLMI | Lebanon | 11 | (0, 20) | 1 | (0, 1) | 22 | (0, 67) |
| AfrLMI | Lesotho | 344 | (154, 462) | 194 | (66, 292) | 189 | (0, 564) |
| AfrLMI | Liberia | 643 | (253, 862) | 391 | (138, 569) | 449 | (0, 1342) |
| EmrLMI | Libya | 25 | (0, 39) | 2 | (0, 3) | 23 | (0, 70) |
| EurLMI | Lithuania | 1 | (0, 2) |  | (0, 0) | 1 | (0, 2) |
| AfrLMI | Madagascar | 3 005 | (1339, 4163) | 2 482 | (887, 3623) | 2 182 | (0, 6479) |
| AfrLMI | Malawi | 2 573 | (1127, 3462) | 1 115 | (371, 1711) | 1 428 | (0, 4271) |
| WprLMI | Malaysia | 51 | (0, 97) | 7 | (1, 15) | 453 | (0, 1343) |
| SearLMI | Maldives | 1 | (0, 2) | 0 | (0, 0) | 4 | (0, 11) |
| AfrLMI | Mali | 5 321 | (2542, 6974) | 2 963 | (1031, 4385) | 4 489 | (0, 13425) |
| WprLMI | Marshall Islands | 2 | (0, 3) | 1 | (0, 1) | 2 | (0, 5) |
| AfrLMI | Mauritania | 682 | (328, 898) | 388 | (138, 575) | 474 | (0, 1417) |
| AfrLMI | Mauritius | 7 | (0, 12) | 1 | (0, 2) | 4 | (0, 13) |
| AmrLMI | Mexico | 729 | (0, 1246) | 172 | (51, 295) | 638 | (0, 1974) |
| WprLMI | Micronesia | 4 | (1, 6) | 3 | (1, 6) | 3 | (0, 10) |
| WprLMI | Mongolia | 44 | (16, 65) | 18 | (5, 31) | 37 | (0, 110) |
| EurLMI | Montenegro | 0 | (0, 0) | 0 | (0, 0) | 0 | (0, 1) |
| EmrLMI | Morocco | 693 | (211, 978) | 134 | (38, 237) | 361 | (0, 1086) |
| AfrLMI | Mozambique | 6 008 | (2820, 7889) | 3 545 | (1235, 5187) | 3 497 | (0, 10458) |
| SearLMI | Myanmar | 3 280 | (900, 4790) | 866 | (243, 1491) | 2 272 | (0, 6896) |
| AfrLMI | Namibia | 126 | (48, 174) | 75 | (25, 114) | 93 | (0, 277) |
| WprLMI | Nauru | 0 | (0, 0) | 0 | (0, 0) | 0 | (0, 1) |
| SearLMI | Nepal | 2 084 | (584, 2949) | 1 187 | (408, 1790) | 1 829 | (0, 5458) |
| AmrLMI | Nicaragua | 101 | (27, 150) | 52 | (16, 84) | 95 | (0, 295) |
| AfrLMI | Niger | 6 802 | (3258, 8989) | 4 402 | (1563, 6387) | 3 384 | (0, 10118) |
| AfrLMI | Nigeria | 51 889 | (24168, 68511) | 27 077 | (9350, 40439) | 31 218 | (0, 93357) |
| WprLMI | Niue | 0 | (0, 0) |  | (0, 0) |  | (0, 0) |
| EmrLMI | Pakistan | 22 046 | (7961, 30473) | 10 635 | (3508, 16382) | 14 833 | (0, 44595) |
| WprLMI | Palau | 0 | (0, 0) | 0 | (0, 0) | 0 | (0, 1) |
| AmrLMI | Panama | 79 | (0, 138) | 30 | (9, 52) | 54 | (0, 167) |
| WprLMI | Papua New Guinea | 401 | (175, 574) | 360 | (124, 541) | 296 | (0, 877) |
| AmrLMI | Paraguay | 83 | (11, 128) | 25 | (8, 44) | 55 | (0, 170) |
| AmrLMI | Peru | 100 | (24, 166) | 96 | (30, 160) | 235 | (0, 700) |
| WprLMI | Philippines | 2 794 | (854, 4038) | 801 | (245, 1355) | 2 644 | (0, 7842) |
| EurLMI | Republic of Moldova | 2 | (1, 3) |  | (0, 1) | 1 | (0, 2) |
| EurLMI | Romania | 8 | (2, 12) | 2 | (0, 3) | 6 | (0, 16) |
| EurLMI | Russian Federation | 453 | (0, 732) | 145 | (42, 256) | 618 | (0, 2086) |
| AfrLMI | Rwanda | 1 249 | (528, 1736) | 507 | (154, 833) | 1 643 | (0, 4914) |
| AmrLMI | Saint Lucia | 1 | (0, 1) | 0 | (0, 1) | 2 | (0, 7) |
| AmrLMI | Saint Vincent and the Grenadines | 0 | (0, 1) | 0 | (0, 0) | 1 | (0, 4) |
| WprLMI | Samoa | 3 | (0, 6) | 1 | (0, 1) | 3 | (0, 9) |
| AfrLMI | Sao Tome and Principe | 15 | (7, 21) | 9 | (3, 14) | 18 | (0, 54) |
| AfrLMI | Senegal | 2 222 | (1060, 2972) | 970 | (316, 1508) | 1 251 | (0, 3760) |
| EurLMI | Serbia | 14 | (0, 24) | 1 | (0, 1) | 12 | (0, 37) |
| AfrLMI | Seychelles | 2 | (0, 3) | 0 | (0, 0) | 5 | (0, 16) |
| AfrLMI | Sierra Leone | 3 310 | (1573, 4313) | 2 025 | (724, 2926) | 1 741 | (0, 5207) |
| WprLMI | Solomon Islands | 24 | (8, 36) | 23 | (8, 35) | 9 | (0, 28) |
| EmrLMI | Somalia | 6 013 | (2540, 8096) | 3 334 | (1170, 4985) | 3 104 | (0, 9331) |
| AfrLMI | South Africa | 3 812 | (657, 5665) | 1 038 | (311, 1762) | 2 934 | (0, 8775) |
| EmrLMI | South Sudan | 3 247 | (1457, 4328) | 1 778 | (546, 2913) | 2 274 | (0, 6801) |
| SearLMI | Sri Lanka | 421 | (129, 620) | 68 | (17, 133) | 476 | (0, 1445) |
| EmrLMI | Sudan | 7 566 | (3754, 9989) | 4 457 | (1524, 6651) | 4 498 | (0, 13522) |
| AmrLMI | Suriname | 2 | (0, 4) | 1 | (0, 1) | 6 | (0, 17) |
| AfrLMI | Swaziland | 179 | (89, 237) | 66 | (21, 105) | 116 | (0, 346) |
| EmrLMI | Syria | 248 | (0, 395) | 18 | (5, 34) | 160 | (0, 481) |
| EurLMI | Tajikistan | 235 | (92, 361) | 37 | (6, 78) | 198 | (0, 591) |
| SearLMI | Thailand | 833 | (215, 1218) | 38 | (7, 81) | 1 563 | (0, 4656) |
| EurLMI | TFYR Macedonia | 1 | (0, 1) | 0 | (0, 0) | 0 | (0, 1) |
| SearLMI | Timor-Leste | 34 | (14, 52) | 48 | (16, 75) | 79 | (0, 241) |
| AfrLMI | Togo | 1 495 | (702, 1964) | 922 | (331, 1340) | 880 | (0, 2633) |
| WprLMI | Tonga | 2 | (0, 4) | 1 | (0, 1) | 2 | (0, 6) |
| EmrLMI | Tunisia | 52 | (0, 82) | 7 | (2, 12) | 56 | (0, 169) |
| EurLMI | Turkey | 286 | (0, 532) | 50 | (14, 93) | 283 | (0, 844) |
| EurLMI | Turkmenistan | 184 | (64, 266) | 13 | (4, 23) | 95 | (0, 284) |
| WprLMI | Tuvalu | 0 | (0, 0) | 0 | (0, 0) | 0 | (0, 1) |
| AfrLMI | Uganda | 6 509 | (2916, 8764) | 3 615 | (1246, 5452) | 5 564 | (0, 16540) |
| EurLMI | Ukraine | 91 | (13, 145) | 9 | (2, 17) | 304 | (0, 906) |
| AfrLMI | Un. Rep. Tanzania | 7 130 | (3264, 9666) | 5 275 | (1884, 7652) | 5 208 | (0, 15574) |
| AmrLMI | Uruguay | 19 | (0, 34) | 0 | (0, 1) | 9 | (0, 34) |
| EurLMI | Uzbekistan | 131 | (49, 225) | 48 | (5, 108) | 532 | (0, 1586) |
| WprLMI | Vanuatu | 11 | (3, 15) | 4 | (1, 7) | 4 | (0, 10) |
| AmrLMI | Venezuela | 200 | (0, 342) | 45 | (13, 80) | 190 | (0, 587) |
| WprLMI | Viet Nam | 321 | (82, 541) | 488 | (149, 808) | 2 031 | (0, 6024) |
| EmrLMI | Yemen | 1 888 | (850, 2531) | 704 | (224, 1137) | 1 648 | (0, 4953) |
| AfrLMI | Zambia | 2 079 | (952, 2765) | 986 | (328, 1504) | 2 005 | (0, 5996) |
| AfrLMI | Zimbabwe | 2 126 | (939, 2882) | 1 046 | (340, 1622) | 535 | (0, 1601) |
| World |  | 502 061 | (217119, 671945) | 280 443 | (95699, 417482) | 296 860 | (0, 885355) |

AfrLMI: Africa region low and middle income countries; AfrHI: Africa region high income countries; AmrLMI: America region low and middle income countries; EmrLMI : Eastern Mediterranean region low and middle income countries; EurLMI: Europe region low and middle income countries; SearLMI : South East Asia region low and middle income countries; WprLMI: Western Pacific low and middle income countries; DPR Korea: Democratic People’s Republic of Korea; DR Congo: Democratic Republic of Congo; Lao PDR: Lao People's Democratic Republic; TFYR Macedonia: The former Yugoslav Republic of Macedonia; Un. Rep. Tanzania: United Republic of Tanzania.

a. Equatorial Guinea has been included despite being classified as high-income countries in 2012 for the purpose of this analysis.

b. World Bank Income classification, July 2012 (The World Bank 2012).

Supplemental Material, Table 2. Deaths estimates attributable to the combined inadequate water and sanitation, and to the combined inadequate water, sanitation and hygiene in low and middle-income countries ^a^, for the year 2012, by country.

|  |  | **Water and sanitation deaths** | | **Water, sanitation and hygiene deaths** | |
| --- | --- | --- | --- | --- | --- |
| **Region^b^** | **Country** | **Mean** | **(95% CI)** | **Mean** | **(95% CI)** |
| EmrLMI | Afghanistan | 8 296 | (5770, 10701) | 9 867 | (6466, 12666) |
| EurLMI | Albania | 5 | (3, 6) | 7 | (2, 11) |
| AfrLMI | Algeria | 616 | (399, 829) | 911 | (325, 1408) |
| AfrLMI | Angola | 18 385 | (13055, 23446) | 22 316 | (14605, 28910) |
| AmrLMI | Anitgua and Barbuda | 0 | (0, 0) | 1 | (0, 3) |
| AmrLMI | Argentina | 154 | (75, 234) | 265 | (35, 458) |
| EurLMI | Armenia | 21 | (12, 31) | 32 | (6, 53) |
| EurLMI | Azerbaijan | 116 | (73, 159) | 195 | (60, 319) |
| SearLMI | Bangladesh | 7 236 | (4209, 10233) | 8 950 | (4988, 12175) |
| EurLMI | Belarus | 11 | (6, 16) | 19 | (3, 33) |
| AmrLMI | Belize | 2 | (2, 3) | 3 | (1, 5) |
| AfrLMI | Benin | 2 607 | (1861, 3277) | 3 063 | (2070, 3874) |
| SearLMI | Bhutan | 38 | (24, 50) | 51 | (25, 74) |
| AmrLMI | Bolivia | 526 | (361, 681) | 708 | (345, 1016) |
| EurLMI | Bosnia and Herzegovina | 2 | (1, 3) | 3 | (1, 4) |
| AfrLMI | Botswana | 137 | (95, 176) | 183 | (86, 264) |
| AmrLMI | Brazil | 1 396 | (862, 1928) | 2 141 | (609, 3448) |
| EurLMI | Bulgaria | 3 | (2, 5) | 6 | (1, 11) |
| AfrLMI | Burkina Faso | 5 335 | (3753, 6832) | 6 338 | (4207, 8064) |
| AfrLMI | Burundi | 5 234 | (3535, 6866) | 6 325 | (4067, 8236) |
| WprLMI | Cambodia | 621 | (411, 820) | 817 | (454, 1130) |
| AfrLMI | Cameroon | 7 053 | (4809, 9107) | 8 547 | (5454, 11169) |
| AfrLMI | Cape Verde | 18 | (13, 22) | 22 | (13, 29) |
| AfrLMI | Central African Republic | 3 865 | (2631, 5017) | 4 566 | (2978, 5839) |
| AfrLMI | Chad | 9 370 | (6611, 11809) | 10 961 | (7568, 13831) |
| AmrLMI | Chile | 55 | (30, 81) | 76 | (12, 134) |
| WprLMI | China | 3 145 | (1989, 4283) | 4 745 | (1922, 7279) |
| AmrLMI | Colombia | 193 | (124, 262) | 292 | (87, 465) |
| AfrLMI | Comoros | 161 | (108, 210) | 198 | (121, 264) |
| AfrLMI | Congo | 1 713 | (1232, 2170) | 2 045 | (1324, 2635) |
| WprLMI | Cook Islands | 0 | (0, 0) | 0 | (0, 1) |
| AmrLMI | Costa Rica | 19 | (10, 28) | 31 | (6, 53) |
| AfrLMI | Côte d'Ivoire | 6 923 | (5052, 8646) | 8 303 | (5360, 10619) |
| AmrLMI | Cuba | 54 | (34, 73) | 82 | (26, 130) |
| SearLMI | DPR Korea | 198 | (117, 275) | 332 | (90, 546) |
| AfrLMI | DR Congo | 57 048 | (39763, 73276) | 67 827 | (45029, 86810) |
| EmrLMI | Djibouti | 170 | (123, 216) | 216 | (116, 301) |
| AmrLMI | Dominica | 0 | (0, 0) | 0 | (0, 1) |
| AmrLMI | Dominican Republic | 129 | (88, 170) | 190 | (81, 286) |
| AmrLMI | Ecuador | 164 | (105, 225) | 255 | (78, 407) |
| EmrLMI | Egypt | 801 | (434, 1173) | 1 208 | (228, 2011) |
| AmrLMI | El Salvador | 103 | (72, 135) | 146 | (62, 219) |
| AfrHI | Equatorial Guinea | 336 | (223, 443) | 411 | (253, 539) |
| AfrLMI | Eritrea | 1 735 | (1234, 2188) | 2 040 | (1351, 2576) |
| AfrLMI | Ethiopia | 22 562 | (15707, 28506) | 26 088 | (17974, 32721) |
| WprLMI | Fiji | 15 | (9, 21) | 25 | (7, 42) |
| AfrLMI | Gabon | 365 | (256, 467) | 453 | (268, 606) |
| AfrLMI | Gambia | 298 | (199, 389) | 372 | (213, 503) |
| EurLMI | Georgia | 5 | (3, 7) | 8 | (3, 12) |
| AfrLMI | Ghana | 4 031 | (2759, 5195) | 4 763 | (3102, 6075) |
| AmrLMI | Grenada | 1 | (0, 1) | 1 | (0, 1) |
| AmrLMI | Guatemala | 870 | (574, 1159) | 1 296 | (439, 2024) |
| AfrLMI | Guinea | 3 799 | (2615, 4887) | 4 506 | (2955, 5762) |
| AfrLMI | Guinea-Bissau | 664 | (463, 851) | 778 | (516, 983) |
| AmrLMI | Guyana | 22 | (14, 29) | 30 | (12, 45) |
| AmrLMI | Haiti | 2 343 | (1572, 3021) | 2 790 | (1777, 3599) |
| AmrLMI | Honduras | 407 | (265, 546) | 595 | (222, 919) |
| SearLMI | India | 271 328 | (172527, 362268) | 334 778 | (197825, 446149) |
| SearLMI | Indonesia | 4 742 | (2625, 6832) | 8 815 | (2296, 15021) |
| EmrLMI | Iran | 459 | (259, 656) | 690 | (141, 1140) |
| EmrLMI | Iraq | 896 | (602, 1191) | 1 256 | (491, 1892) |
| AmrLMI | Jamaica | 33 | (21, 45) | 50 | (19, 78) |
| EmrLMI | Jordan | 40 | (22, 58) | 66 | (13, 110) |
| EurLMI | Kazakhstan | 85 | (44, 128) | 168 | (27, 296) |
| AfrLMI | Kenya | 10 984 | (7741, 14110) | 13 497 | (8465, 17669) |
| WprLMI | Kiribati | 12 | (8, 15) | 16 | (8, 22) |
| EurLMI | Kyrgyzstan | 64 | (38, 89) | 97 | (34, 155) |
| WprLMI | Lao PDR | 602 | (368, 825) | 909 | (377, 1388) |
| EurLMI | Latvia | 1 | (0, 1) | 1 | (0, 2) |
| EmrLMI | Lebanon | 12 | (6, 18) | 18 | (3, 31) |
| AfrLMI | Lesotho | 468 | (323, 606) | 566 | (358, 735) |
| AfrLMI | Liberia | 877 | (600, 1125) | 1 028 | (675, 1300) |
| EmrLMI | Libya | 26 | (16, 36) | 38 | (12, 60) |
| EurLMI | Lithuania | 1 | (1, 2) | 2 | (0, 3) |
| AfrLMI | Madagascar | 4 748 | (3212, 6101) | 5 840 | (3696, 7696) |
| AfrLMI | Malawi | 3 273 | (2164, 4324) | 4 008 | (2484, 5283) |
| WprLMI | Malaysia | 56 | (28, 84) | 110 | (15, 196) |
| SearLMI | Maldives | 1 | (1, 2) | 2 | (1, 3) |
| AfrLMI | Mali | 7 139 | (5063, 9080) | 8 444 | (5577, 10714) |
| WprLMI | Marshall Islands | 3 | (1, 4) | 4 | (1, 6) |
| AfrLMI | Mauritania | 922 | (669, 1157) | 1 084 | (723, 1374) |
| AfrLMI | Mauritius | 7 | (4, 11) | 11 | (2, 19) |
| AmrLMI | Mexico | 859 | (527, 1184) | 1 330 | (373, 2146) |
| WprLMI | Micronesia | 7 | (4, 10) | 10 | (5, 14) |
| WprLMI | Mongolia | 58 | (34, 82) | 85 | (39, 127) |
| EurLMI | Montenegro | 0 | (0, 0) | 0 | (0, 1) |
| EmrLMI | Morocco | 785 | (554, 1004) | 1 048 | (516, 1504) |
| AfrLMI | Mozambique | 8 118 | (5833, 10303) | 9 499 | (6504, 11925) |
| SearLMI | Myanmar | 3 905 | (2046, 5717) | 5 394 | (2542, 7855) |
| AfrLMI | Namibia | 178 | (124, 227) | 219 | (133, 292) |
| WprLMI | Nauru | 0 | (0, 0) | 0 | (0, 1) |
| SearLMI | Nepal | 2 878 | (1768, 3909) | 3 522 | (2105, 4703) |
| AmrLMI | Nicaragua | 140 | (97, 179) | 187 | (95, 268) |
| AfrLMI | Niger | 9 478 | (6727, 11927) | 11 081 | (7665, 13964) |
| AfrLMI | Nigeria | 68 254 | (46724, 87438) | 80 968 | (53271, 103585) |
| WprLMI | Niue | 0 | (0, 0) | 0 | (0, 0) |
| EmrLMI | Pakistan | 28 992 | (19514, 38044) | 36 127 | (21780, 48263) |
| WprLMI | Palau | 0 | (0, 0) | 1 | (0, 1) |
| AmrLMI | Panama | 103 | (65, 140) | 153 | (48, 241) |
| WprLMI | Papua New Guinea | 669 | (443, 881) | 858 | (508, 1166) |
| AmrLMI | Paraguay | 103 | (70, 134) | 147 | (62, 222) |
| AmrLMI | Peru | 187 | (114, 257) | 352 | (90, 602) |
| WprLMI | Philippines | 3 394 | (2047, 4690) | 4 723 | (2246, 6894) |
| EurLMI | Republic of Moldova | 2 | (1, 3) | 3 | (1, 5) |
| EurLMI | Romania | 9 | (6, 12) | 14 | (5, 22) |
| EurHI | Russian Federation | 560 | (362, 748) | 719 | (271, 1097) |
| AfrLMI | Rwanda | 1 606 | (1000, 2194) | 2 119 | (1132, 2961) |
| AmrLMI | Saint Lucia | 1 | (1, 1) | 1 | (1, 2) |
| AmrLMI | Saint Vincent & the Grenadines | 1 | (0, 1) | 1 | (0, 1) |
| WprLMI | Samoa | 4 | (2, 5) | 7 | (1, 12) |
| AfrLMI | Sao Tome and Principe | 21 | (15, 27) | 26 | (17, 33) |
| AfrLMI | Senegal | 2 844 | (2066, 3587) | 3 482 | (2178, 4570) |
| EurLMI | Serbia | 15 | (8, 21) | 25 | (5, 43) |
| AfrLMI | Seychelles | 2 | (1, 3) | 3 | (1, 4) |
| AfrLMI | Sierra Leone | 4 504 | (3220, 5655) | 5 231 | (3659, 6522) |
| WprLMI | Solomon Islands | 42 | (27, 56) | 56 | (31, 78) |
| EmrLMI | Somalia | 8 047 | (5529, 10358) | 9 597 | (6366, 12358) |
| AfrLMI | South Africa | 4 564 | (3158, 5897) | 6 258 | (2854, 9229) |
| EmrLMI | South Sudan | 4 249 | (2952, 5431) | 5 217 | (3366, 6720) |
| SearLMI | Sri Lanka | 481 | (240, 718) | 705 | (284, 1083) |
| EmrLMI | Sudan | 10 310 | (7532, 12879) | 12 309 | (8235, 15648) |
| AmrLMI | Suriname | 3 | (2, 4) | 4 | (1, 6) |
| AfrLMI | Swaziland | 222 | (157, 281) | 276 | (169, 368) |
| EmrLMI | Syria | 260 | (158, 362) | 379 | (108, 599) |
| EurLMI | Tajikistan | 264 | (154, 372) | 579 | (87, 1043) |
| SearLMI | Thailand | 879 | (439, 1317) | 1 241 | (489, 1888) |
| EurLMI | TFYR Macedonia | 1 | (0, 1) | 1 | (0, 1) |
| SearLMI | Timor-Leste | 76 | (44, 104) | 114 | (49, 174) |
| AfrLMI | Togo | 2 043 | (1457, 2577) | 2 377 | (1651, 2972) |
| WprLMI | Tonga | 3 | (2, 4) | 5 | (1, 8) |
| EmrLMI | Tunisia | 57 | (38, 77) | 82 | (28, 128) |
| EurLMI | Turkey | 329 | (177, 478) | 560 | (88, 961) |
| EurLMI | Turkmenistan | 195 | (121, 268) | 299 | (118, 466) |
| WprLMI | Tuvalu | 0 | (0, 0) | 0 | (0, 1) |
| AfrLMI | Uganda | 8 857 | (6013, 11446) | 10 816 | (6831, 14135) |
| EurLMI | Ukraine | 103 | (55, 150) | 169 | (46, 283) |
| AfrLMI | Un. Rep. Tanzania | 10 610 | (7287, 13526) | 12 913 | (8330, 16641) |
| AmrHI | Uruguay | 19 | (9, 29) | 27 | (1, 49) |
| EurLMI | Uzbekistan | 179 | (86, 275) | 657 | (0, 1371) |
| WprLMI | Vanuatu | 14 | (8, 19) | 17 | (9, 24) |
| AmrLMI | Venezuela | 236 | (151, 323) | 364 | (100, 589) |
| WprLMI | Viet Nam | 783 | (406, 1145) | 1 772 | (255, 3259) |
| EmrLMI | Yemen | 2 301 | (1643, 2932) | 2 945 | (1742, 3961) |
| AfrLMI | Zambia | 2 698 | (1862, 3492) | 3 308 | (2107, 4336) |
| AfrLMI | Zimbabwe | 2 821 | (1958, 3620) | 3 539 | (2137, 4724) |
| World |  | 684 456 | (580456, 780463) | 841 818 | (699059, 963626) |

AfrLMI: Africa region low and middle income countries; AfrHI: Africa region high income countries; AmrLMI: America region low and middle income countries; EmrLMI : Eastern Mediterranean region low and middle income countries; EurLMI: Europe region low and middle income countries; SearLMI : South East Asia region low and middle income countries; WprLMI: Western Pacific low and middle income countries; DPR Korea: Democratic People’s Republic of Korea; DR Congo: Democratic Republic of Congo; Lao PDR: Lao People's Democratic Republic; TFYR Macedonia: The former Yugoslav Republic of Macedonia; Un. Rep. Tanzania: United Republic of Tanzania.

a. Equatorial Guinea has been included despite being classified as high-income countries in 2012 for the purpose of this analysis.

b. World Bank Income classification, July 2012 (The World Bank 2012).
